# Interferon-alpha2 treatment of patients with polycythemia vera and related neoplasms favorably impacts deregulation of oxidative stress genes and antioxidative defense mechanisms

**DOI:** 10.1371/journal.pone.0270669

**Published:** 2022-06-30

**Authors:** Vibe Skov, Mads Thomassen, Lasse Kjær, Christina Ellervik, Morten Kranker Larsen, Trine Alma Knudsen, Torben A. Kruse, Hans C. Hasselbalch

**Affiliations:** 1 Department of Hematology, Zealand University Hospital, Roskilde, Denmark; 2 Department of Clinical Genetics, Odense University Hospital, Odense, Denmark; 3 Department of Laboratory Medicine, Boston Children’s Hospital, Harvard Medical School, Boston, Massachusetts, United States of America; Emory University, UNITED STATES

## Abstract

Chronic inflammation is considered a major driving force for clonal expansion and evolution in the Philadelphia-negative myeloproliferative neoplasms, which include essential thrombocythemia, polycythemia vera and primary myelofibrosis (MPNs). One of the key mutation drivers is the *JAK2V617F* mutation, which has been shown to induce the generation of reactive oxygen species (ROS). Using whole blood gene expression profiling, deregulation of several oxidative stress and anti-oxidative defense genes has been identified in MPNs, including significant downregulation of *TP53*, the *NFE2L2* or *NRF2* genes. These genes have a major role for maintaining genomic stability, regulation of the oxidative stress response and in modulating migration or retention of hematopoietic stem cells. Therefore, their deregulation might give rise to increasing genomic instability, increased chronic inflammation and disease progression with egress of hematopoietic stem cells from the bone marrow to seed in the spleen, liver and elsewhere. Interferon-alpha2 (rIFNα) is increasingly being recognized as the drug of choice for the treatment of patients with MPNs. Herein, we report the first gene expression profiling study on the impact of rIFNα upon oxidative stress and antioxidative defense genes in patients with MPNs (n = 33), showing that rIFNα downregulates several upregulated oxidative stress genes and upregulates downregulated antioxidative defense genes. Treatment with rIFNα induced upregulation of 19 genes in ET and 29 genes in PV including *CXCR4* and *TP53*. In conclusion, this rIFNα- mediated dampening of genotoxic damage to hematopoietic cells may ultimately diminish the risk of additional mutations and accordingly clonal evolution and disease progression towards myelofibrotic and leukemic transformation.

## Introduction

The Philadelphia-negative myeloproliferative neoplasms (MPNs) include essential thrombocythemia (ET), polycythemia vera (PV) and primary myelofibrosis (PMF), which in the large majority are acquired hematopoietic stem cell diseases, arising and driven by somatic stem cell mutations (*JAK2V617F*, *CALR*, *MPL*). Additional mutations are key determinants for clonal expansion and evolution [1, 2]. Chronic inflammation is today considered a major driving force for clonal expansion and evolution in the biological continuum from the early cancer stages (ET, PV) towards the advanced myelofibrosis stage with bone marrow failure, huge splenomegaly and ultimately leukemic transformation [3–27]. The *JAK2V617F* mutation has been shown to generate reactive oxygen species (ROS) [28]. Both the *JAK2V617F* mutation and ROS associate with an increased risk of thrombosis [29–33], extracellular neutrophil trap formation (NETosis) [32] ischemic heart disease [34–39], and cancer as well [34, 40, 41]. Most recently, the *CALR* mutation has also been shown to be involved in the development of a chronic inflammatory state, cells harboring the *CALR* mutation exhibiting cell-autonomous activation of the IL-6 pathway [42].

By whole blood transcriptional profiling, we have previously identified a massive deregulation of several oxidative stress and antioxidative defense genes in patients with MPNs [43]. Amongst the genes significantly downregulated are *TP53* and the *NFE2L2* or *NRF2* gene, the latter having a key role in the regulation of the oxidative stress response and in modulating both migration and retention of hematopoietic stem cells (HSCs). During MPN-disease progression, the HSC pool is steadily expanding with the egress of CD34+cell from stem cell niches into the circulation. In addition to *NRF2*, several other genes are involved in this process, including *CXCR4*, which is also significantly downregulated in MPNs [43].

Interferon-alpha2 (rIFNα) is increasingly been recognized as a very efficacious and promising treatment modality in the treatment of MPNs [44–60]. Taken into account that chronic inflammation with ROS accumulation has a major role in the pathogenesis of MPNs [3–28], ultimately implying the induction of an altered redox balance of pivotal significance for stem cell mobilization [31, 43], we herein report the first study containing novel information on the impact of rIFNα upon oxidative stress and antioxidative defense genes, showing that rIFNα downregulates several upregulated oxidative stress genes and upregulates downregulated antioxidative defense genes.

## Materials and methods

Nineteen patients with ET, 41 patients with PV, and 9 patients with PMF (data set 1), and 8 patients with ET, 21 patients with PV, and 4 patients with PMF (data set 2) as well as 21 control subjects participated in the study. Patients from data set 2 received monotherapy with rIFNα, in the large majority in a dosage ranging from 45 μg x 1 sc/week to 90 μg x 1 sc/week. When patients initiated IFN-monotherapy, hydroxyurea (n = 18) or anagrelide (n = 6) were discontinued. Patient characteristics are presented in Tables 1 and 2, which have been published previously [43]. The study was approved by the regional ethics committee and was performed in accordance with the declaration of Helsinki. Informed written consent was obtained from all subjects before participation.

**Table 1:** Patient characteristics. Data set 1.

	No	Gender	Age	Disease duration (months)	*JAK2*	*JAK2*	Therapy	Thrombosis (+/-)
					*V617F*	*V617F*		
		(m/f)	(years)		(+/-)	(%)		
ET	19	9/10	60 (35–87)	40 (15–278)	9/10	23 (1–55)	HU = 10	9/10
							rIFNα = 3	
							ANA = 5	
							BU = 1	
PV	41	21/20	69 (35–85)	39 (2–171)	40/1	37 (28–48)	HU = 26	19/22
							rIFNα = 5	
							ANA = 1	
							BU = 6	
							None = 3	
PMF	9	3/6	68 (53–74)	31 (11–204)	2/7	59	HU = 1	1/8
							None = 8	

Age: Median and range; Disease duration: Median and range; *JAK2V617F* %: median and 95% confidence interval; HU = hydroxyurea; rIFNα = interferon-alpha2; ANA = anagrelide; BU = busulfan.

**Table 2:** Patient characteristics at baseline. Data set 2.

	No	Gender	Age	*JAK2*	*JAK2*	Therapy at baseline
				*V617F*	*V617F*	
		(m/f)	(years)	(+/-)	(%)	
ET	8	4/4	57 (45–66)	6/2	14 (1–48)	HU = 5
						None = 3
PV	21	10/11	62 (26–69)	21/0	20 (10–79)	HU = 14
						None = 5
						ANA = 2
PMF	4	2/2	68 (55–73)	4/0	30 (6–92)	None = 4

Age: Median and range; *JAK2V617F* %: median and 95% confidence interval; HU = hydroxyurea; ANA = anagrelide.

In data set 1 and 2, gene expression profiles from patients with ET, PV or PMF were compared with 21 control subjects. In data set 2, gene expression profiling of patients with ET, PV and PMF was performed at baseline and after 3 months of treatment with rIFNα. The effect of rIFNα on transcriptional changes in patients from data set 2 was tested using gene expression profiling of 38.500 genes. Whole blood was collected in Paxgene tubes (Preanalytix, Hombrechtikon, Switzerland), stored at room temperature for 24 h, then at -20°C for minimum one day, and finally transferred to a -80°C freezer. The Paxgene Blood RNA kit (Qiagen, Franklin Lakes, NJ, USA) was used to extract total RNA from each sample. The quantity and quality of RNA were tested with NanoDrop spectrophotometer ND-8000 (NanoDrop Technologies) and Agilent 2100 Bioanalyzer (Agilent Technologies, Palo Alto, CA), respectively. Three hundred ng of purified total RNA were converted to biotin-labeled aRNA using the Message-AmpTM III RNA amplification kit (Ambion, Austin, TX). Amplified RNA was hybridized to Affymetrix HG-U133 2.0 Plus microarrays and scanned with the Affymetrix GeneChip Scanner 3000-7G (Affymetrix, Santa Clara, CA).

The R statistical software [61] and the “robust multi-array average method” (RMA) [62] from the Bioconductor package “affy” were applied to perform background correction, normalization, and gene expression index calculation of probe intensities. Only perfect match probes were used for data analysis. Differences in gene expression between patients and controls (data set 1 and 2) as well as in patients before and after 3 months of treatment with rIFNα (data set 2) were calculated using the regularized t-test from the Bioconductor package “limma” for unpaired and paired data, respectively. Data set 1 has been performed and analyzed as a hypothesis generating study, therefore we applied an FDR value<0.05 to control for multiple hypothesis testing. Data set 2 was performed later and hypotheses generated in data set 1 were tested in data set 2. Thus, a p value <0.05 was applied as a cut off for significance in data set 2. Data are available from Gene Expression Omnibus (http://www.ncbi.nlm.nih.gov/geo; accession no. GSE57793).

## Results

Single gene analysis of 148 genes found to be included in previous studies focusing on deregulation of oxidative stress and antioxidative defense genes in various diseases were chosen for further analysis. Since our previous study of gene expression profiling of oxidative stress genes in patients with MPNs compared to control subjects [43], 6 genes have been added to the panel of oxidative stress and antioxidative defense genes, totaling 154 genes. In response to treatment with rIFNα, 19 genes were upregulated in ET including *CXCR4* and *TP53*, and 11 genes were downregulated including *FOXO3*, *PRDX2*, and *PRDX6* (Table 3).

**Table 3:** The significantly most up- or downregulated genes in patients with ET during treatment with rIFNα (data set 2). In addition, data from the same patients compared to controls (data set 2) and another cohort (data set 1) are presented.

	Data set 1		Data set 2		Data set 2	
	Ctrl vs baseline		Ctrl vs baseline		Baseline vs rIFNα	
**Upregulated genes**	**ET**		**ET**		**ET**	
*Symbol*	*FC*	*FDR*	*FC*	*Pvalue*	*FC*	*Pvalue*
MSRA	-1,2	0,1	2,6	3,7E-09	1,9	0,0002
DEFA4	1,5	0,2	1,8	0,04	1,7	0,02
DEFA1	1,1	0,8	1,6	0,09	1,7	0,047
PRDX4	-1,02	0,9	1,3	0,09	1,3	0,004
PRPS2	-1,1	0,3	1,1	0,2	1,3	0,01
DEFT1P	1,1	0,4	1,2	0,006	1,2	0,04
AKR1A1	-1,1	0,2	1,3	0,005	1,2	0,02
HSPA1A	-1,8	0,002	1,2	0,02	1,2	0,02
TP53	-1,4	0,0003	-1,2	0,009	1,2	0,006
GLRX	-1,0	0,8	1,1	0,3	1,2	0,01
PRDX1	-1,2	0,02	1,02	0,9	1,2	0,04
DEFB106A	1,1	0,2	1,1	0,4	1,2	0,04
MPO	1,3	0,005	1,2	0,009	1,2	0,02
SELS	-1,4	0,0006	-1,03	0,8	1,2	0,04
PFKP	-1,1	2,0E-01	1,1	0,1	1,2	0,04
CXCR4	-2,1	3,7E-09	-1,1	0,2	1,2	0,04
MGST2	-1,0	0,9	1,03	0,7	1,2	0,04
PFKM	-1,1	1,7E-01	1,3	5,3E-06	1,1	0,01
ALDOA	-1,3	0,002	1,1	0,2	1,1	0,03
**Downregulated genes**	**ET**		**ET**		**ET**	
*Symbol*	*FC*	*FDR*	*FC*	*Pvalue*	*FC*	*Pvalue*
IPCEF1	-1,1	0,2	1,03	0,9	-1,9	0,001
PRDX2	1,6	0,01	1,01	1,0	-1,6	0,008
CAT	-2,4	1,3E-08	-2,0	1,6E-06	-1,5	8,8E-05
FOXO3	1,2	0,2	1,5	0,02	-1,5	0,0007
CSDE1	-1,5	1,3E-05	-1,4	5,3E-08	-1,3	0,0007
SOD2	-1,8	0,003	-1,9	0,005	-1,3	0,03
NQO2	-1,2	3,0E-01	-1,5	0,046	-1,2	0,003
PRDX6	1,3	0,03	1,3	0,03	-1,2	0,02
GSTZ1	1,2	4,2E-04	-1,4	1,8E-06	-1,2	0,008
SRXN1	1,1	0,6	-1,1	0,6	-1,2	0,01
PXDN	1,1	0,1	-1,1	0,02	-1,1	0,03

Ctrl: control; rIFNα: interferon-alpha2; ET: essential thrombocythemia; FC: fold change; FDR: false discovery rate.

In patients with PV, *ATOX1*, *CXCR4*, *SEPP1*, and *TP53* were among the 29 upregulated genes, and *FOXO3* and *PRDX2* were among the 14 downregulated genes (Table 4).

**Table 4:** The significantly most up- or downregulated genes in patients with PV during treatment with rIFNα (data set 2). In addition, data from the same patients compared to controls (data set 2) and another cohort (data set 1) are presented.

	Data set 1		Data set 2		Data set 2	
	Ctrl vs baseline		Ctrl vs baseline		Baseline vs rIFNα	
**Upregulated genes**	**PV**		**PV**		**PV**	
*Symbol*	*FC*	*FDR*	*FC*	*Pvalue*	*FC*	*Pvalue*
MSRA	1,1	0,3	1,5	0,0002	2,2	1,2E-08
DEFA4	1,6	0,06	1,3	0,4	1,5	0,04
DEFA1	1,2	0,5	1,01	1,0	1,5	0,04
TP53	-1,6	3,6E-06	-1,6	2,5E-06	1,3	1,3E-06
PRDX4	-1,1	0,4	-1,1	0,2	1,3	0,0004
CYBB	-1,6	7,3E-06	-1,4	0,0001	1,3	0,0003
MGST3	-1,1	0,3	1,3	0,02	1,3	0,02
PRDX1	-1,2	0,003	-1,2	0,02	1,2	0,0007
ATOX1	1,3	5,2E-06	1,2	7,0E-05	1,2	2,1E-05
PFKP	-1,2	6,8E-03	-1,1	0,06	1,2	0,003
HSPA1A	-1,2	0,2	1,1	0,2	1,2	0,001
CXCR4	-2,0	1,1E-11	-1,1	0,3	1,2	0,007
MPO	1,2	0,006	-1,2	0,002	1,2	1,7E-05
GLRX2	-1,0	1	-1,1	0,09	1,2	0,007
PRPS2	1,0	0,9	-1,1	0,5	1,2	0,03
AKR1A1	-1,1	0,1	-1,02	0,7	1,2	0,0002
GSTM4	-1,2	0,01	-1,3	0,003	1,2	0,003
SEPP1	1,3	0,0001	1,2	0,0004	1,2	0,009
GSR	1,1	0,2	-1,1	0,03	1,2	0,002
GLRX	1,2	0,1	-1,1	0,2	1,2	0,004
NCF1	-1,1	0,2	1,1	0,2	1,2	0,01
SELS	-1,4	5,2E-06	-1,2	0,006	1,1	0,02
GTF2I	-1,6	1,2E-07	-1,04	0,5	1,1	0,002
NUDT1	1,0	4,6E-01	-1,01	0,8	1,1	0,004
DGKK	-1,03	0,5	-1,1	0,07	1,1	0,005
MGST2	-1	0,9	-1,02	0,7	1,1	0,02
MGST1	1,05	0,4	-1,05	0,2	1,1	0,01
PFKM	-1,1	2,8E-01	1,01	0,8	1,1	0,02
TXNRD2	-1,1	0,03	-1	0,9	1,1	0,04
**Downregulated genes**	**PV**		**PV**		**PV**	
*Symbol*	*FC*	*FDR*	*FC*	*Pvalue*	*FC*	*Pvalue*
FOXO3	1,6	6,3E-05	1,6	6,1E-05	-1,5	2,3E-06
NQO2	1,2	0,2	1,5	0,008	-1,5	1,8E-05
CAT	-1,9	6,4E-06	-1,1	0,5	-1,4	0,0002
PRDX2	1,7	0,0004	1,4	0,006	-1,4	0,0009
GSTZ1	1,2	4,8E-05	-1,1	0,006	-1,3	4,3E-06
IPCEF1	-1,05	0,5	1,4	0,1	-1,3	0,01
SOD2	-1,6	0,004	-1,5	0,008	-1,3	6,8E-05
CSDE1	-1,3	4,8E-05	-1,1	0,1	-1,2	0,002
GCLC	1,4	0,008	1,4	0,008	-1,2	0,002
TALDO1	-1,1	1,5E-01	1,2	0,02	-1,2	0,004
SRXN1	1,2	0,05	1,1	0,1	-1,2	0,0003
TKT	-1,6	2,0E-07	-1,1	0,4	-1,2	0,02
PREX1	-1,4	0,001	-1,1	0,1	-1,1	0,03
SQSTM1	1,2	4,6E-06	1,1	0,0002	-1,1	0,005

Ctrl: control; rIFNα: interferon-alpha2; PV: polycythemia vera; FC: fold change; FDR: false discovery rate.

In response to treatment with rIFNα, two genes were upregulated in PMF which were *CYBB* and *MSRA*, and nine genes were downregulated including *PRDX2*, and *FOXO3* (Table 5) (All P<0.05).

**Table 5:** The significantly most up- or downregulated genes in patients with PMF during treatment with rIFNα (data set 2). In addition, data from the same patients compared to controls (data set 2) and another cohort (data set 1) are presented.

	Data set 1		Data set 2		Data set 2	
	Ctrl vs baseline		Ctrl vs baseline		Baseline vs rIFNα	
**Upregulated genes**	**PMF**		**PMF**		**PMF**	
*Symbol*	*FC*	*FDR*	*FC*	*Pvalue*	*FC*	*Pvalue*
MSRA	1,2	0,1	1,4	0,006	1,8	4,9E-05
CYBB	-1,2	0,2	-1,2	0,2	1,2	0,04
**Downregulated genes**	**PMF**		**PMF**		**PMF**	
*Symbol*	*FC*	*FDR*	*FC*	*Pvalue*	*FC*	*Pvalue*
FOXO3	1,7	0,008	1,9	0,0005	-1,7	0,003
PRDX2	4,4	9,1E-06	3,2	2,4E-06	-1,7	0,0008
SOD2	-2,1	0,006	-1,6	0,1	-1,5	0,009
GSTZ1	1,4	2,9E-05	-1,2	0,02	-1,3	0,01
GCLC	2,4	0,0002	2	0,0006	-1,3	0,03
CSDE1	-1,1	0,1	-1,02	0,7	-1,3	0,03
SRXN1	1,2	0,3	1,4	0,01	-1,3	0,01
PDLIM1	1,4	0,1	1,5	0,008	-1,3	0,046
TALDO1	-1,2	0,07	1,1	0,4	-1,2	0,046

Ctrl: control; rIFNα: interferon-alpha2; PMF: primary myelofibrosis; FC: fold change; FDR: false discovery rate.

**Fig 1:**
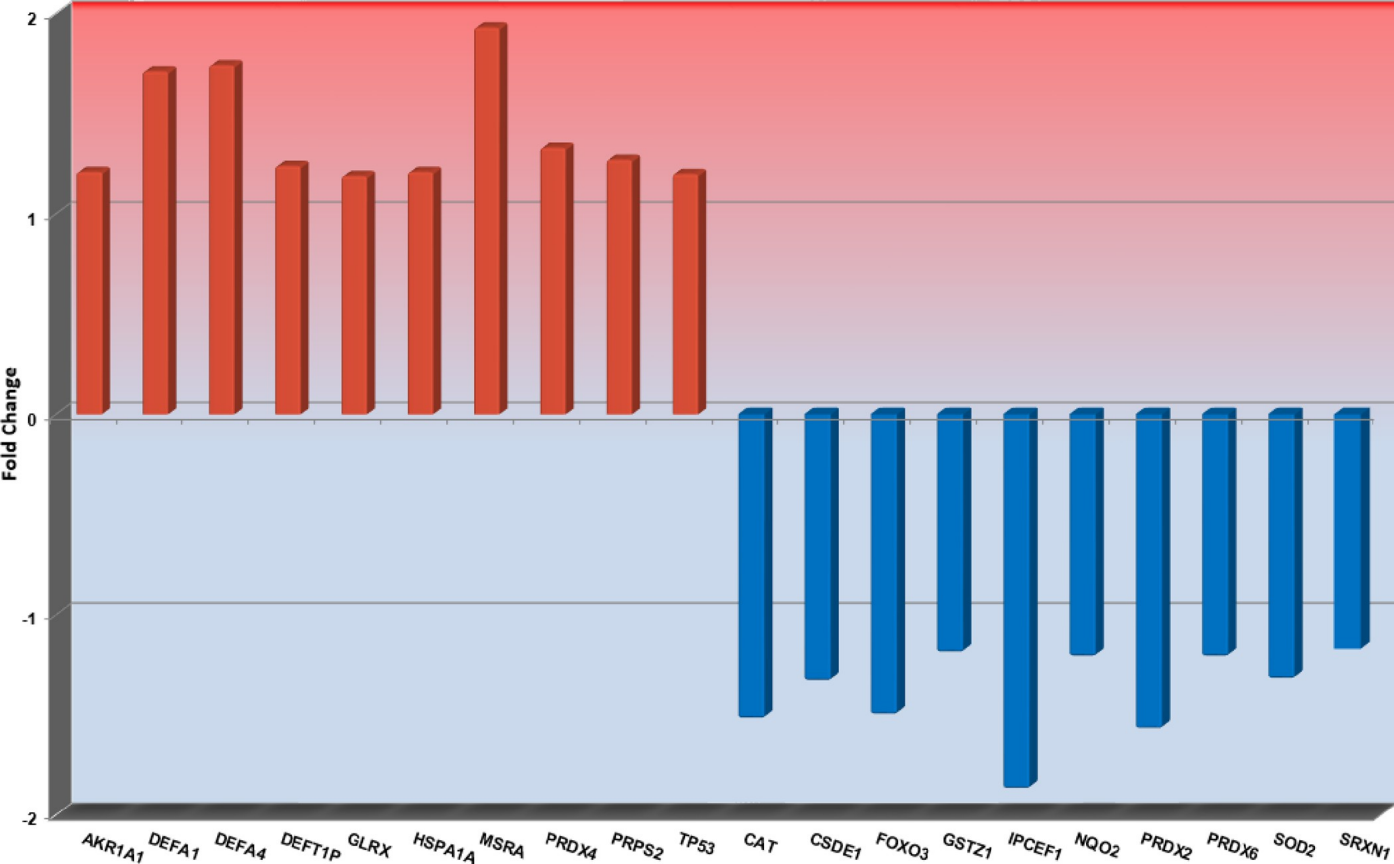
The Top10 most up- or downregulated genes in patients with ET during rIFNα therapy. Fold changes for each gene are shown on the y-axis, and gene names are shown on the x-axis.

**Fig 2:**
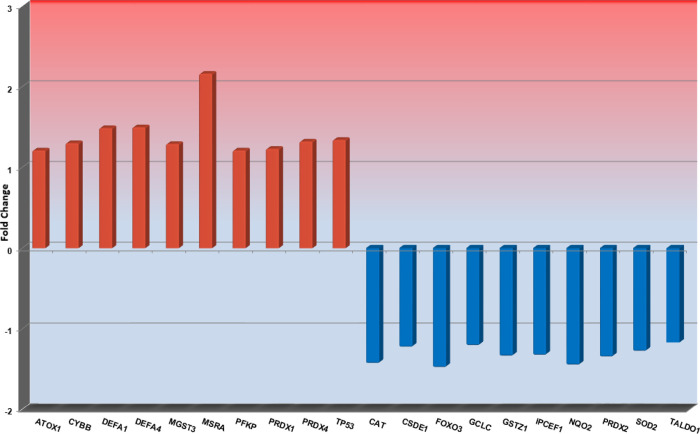
The Top10 most up- or downregulated genes in patients with PV during rIFNα therapy. Fold changes for each gene are shown on the y-axis, and gene names are shown on the x-axis.

**Fig 3:**
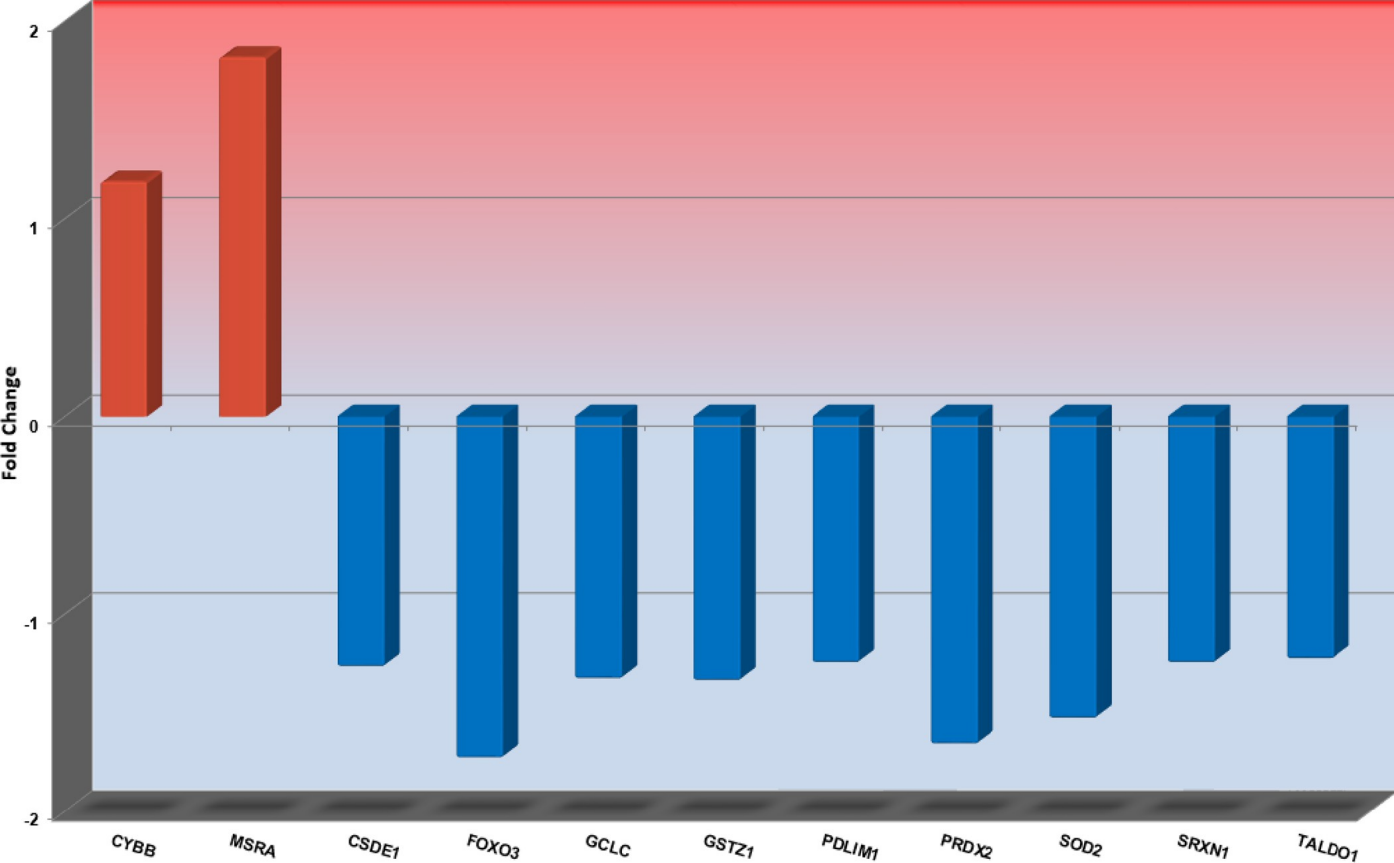
The Top10 most up- or downregulated genes in patients with PMF during rIFNα therapy. Fold changes for each gene are shown on the y-axis, and gene names are shown on the x-axis.

The Top 10 significantly up- or downregulated genes in ET or PV during treatment with rIFNα are shown in Figs 1 and 2, respectively, and the significantly up- or downregulated genes in PMF during rIFNα therapy are shown in Fig 3.

In a previous microarray study (data set 1) [43], we have shown significant downregulation of genes associated with oxidative stress and antioxidative defense including *ATM*, *CYBA*, *NRF2*, *SIRT2*, and *TTN*, and significant upregulation of *AKR1B1*, *CCND1*, *DEFB122*, *GPX8* and *PTGS1* in patients with ET, PV, and PMF compared to controls. These genes were not significantly deregulated during treatment with rIFNα in any of the disease entities. In ET and PV, *CXCR4* and *TP53* were significantly downregulated at baseline and significantly upregulated during treatment with rIFNα. In PV and PMF, *FOXO3* and *GCLC* were significantly upregulated at baseline and significantly downregulated during treatment with rIFNα. In addition, *PRDX2* was significantly upregulated at baseline and significantly downregulated during treatment with rIFNα in all three disease entities. Fold changes and p-values for all 154 oxidative stress and antioxidative defense genes at baseline and during treatment with rIFNα are presented in S1 Table.

## Discussion

In recent years, several studies have substantiated that rIFNα is highly efficacious in the treatment of patients with ET, PV and early hyperproliferative myelofibrosis, implying a normalization of elevated blood cell counts within weeks, a decrease in the *JAK2V617F* allele burden within the first year in most patients [45–60], and in a subset of patients even induction of minimal residual disease as defined by low-burden *JAK2V617F* (< 1%) in concert with normalization of the bone marrow after long-term treatment (> 5 years) [63–67]. The mechanisms of action of rIFNα are pleitropic including upregulation of downregulated HLA-genes [68] and boosting of virtually all immune cells [69–71]. Furthermore, rIFNα has been shown to exhaust and/or deplete malignant stem cells by inducing changes in the cell cycle and apoptosis [50, 51, 57]. Adding these mechanisms, we herein report that rIFNα has a major impact upon deregulated oxidative stress genes and antioxidative defense genes, implying a gene signature that in essence corresponds to decreased oxidative stress and enhancement of antioxidative defense genes. We have previously reported significant deregulation of several oxidative stress and antioxidative defense genes in patients with MPNs compared to control subjects [43]. In regard to *TP53*—being significantly downregulated before rIFNα treatment–this gene was significantly upregulated in ET and PV and no longer deregulated in PMF during rIFNα treatment. Downregulation of *TP53* implies genomic instability due to an increased burden of oxidative stress upon the genome, which accordingly is reduced by rIFNα treatment. Although our genome profile of upregulation of *TP53* strongly argues for enhancement of genomic stability during rIFNα-treatment, this has to be confirmed in protein (e.g. proteomics) and functionality studies, confirming that the function of P53 as a tumor suppressor protein is indeed being improved during rIFNα-treatment of patients with MPNs. *NRF2* is a master regulator of the antioxidant response and has a major role for normal stem cell function [72]. Furthermore, *NRF2* also has a protective role in chronic inflammatory diseases by attenuating the inflammatory state, e.g. in rheumatoid arthritis and atherosclerosis [73]. Thus, our findings of a change in the *NRF2* gene expression from being significantly downregulated before rIFNα exposure to not being deregulated during treatment with rIFNα may imply an improvement in the antioxidative defense mechanisms against increased oxidative stress and accumulation of ROS. These effects may not only dampen the chronic inflammatory drive on the malignant clone, but also potentially enhance the efficacy of immune cells, which are known to be malfunctioning in MPNs [74–76] and negatively impacted by ROS [77, 78]. Since ROS has been shown to decrease interferon production [79], the impact of rIFNα in regulating deregulated oxidative stress and antioxidative defense genes may actually also improve the defense against infections. This is not trivial, since recent studies have documented MPN patients to have an increased morbidity and mortality due to infections [80, 81], which accordingly might not only be explained by impaired functionality of immune cells (e.g. T-cell exhaustion) [77] but also by defective interferon production, mediated by excessive ROS accumulation.

The *CXCR4* gene was significantly downregulated at baseline in ET and PV patients and became significantly upregulated during treatment with rIFNα. *CXCR4* has an important role for homing and retention of hematopoietic stem cells (HSC) [82] and for maintaining HSC quiescence as well [83]. We and others have previously reported *CXCR4* to be significantly downregulated in MPNs, in particular in myelofibrosis [43], consistent with previous studies displaying downregulation of *CXCR4* in CD34+ cells [84]. Accordingly, upregulation of the *CXCR4* gene during rIFNα treatment might prohibit egress of CD34 + positive stem cells from bone marrow niches and accordingly development of extramedullary hematopoiesis in the spleen and liver. Indeed, by significantly upregulating Nrf2 and CXCR4, rIFNα may improve the balance between stem cell quiescence and proliferation, self-renewal and differentiation, and also significantly influencing homing and retention of HSCs in the bone marrow niche. Since excessive ROS accumulation and oxidative stress may also contribute to aberrant DNA methylation [85], which has been reported in patients with MPNs [86], including hypermethylation of the CXCR4 promoter in CD34+ cells in PMF patients, it is intriguing to consider, if rIFNα might actually normalize aberrant DNA methylation in MPNs–a research topic to be pursued in the future.

*PRDX2* (Peroxiredoxin 2) is an antioxidant enzyme that is involved in the scavenging of H_2_O_2_ and ROS, thereby protecting cells from oxidative stress [87]. The *PRDX2* gene has been found to be upregulated in several cancers, and most recently, *PRDX2* has been included in a 6-gene anti-oxidant signature that effectively predicts the prognosis of patients with kidney cancer [88]. Highly interesting, in our patients the *PRDX2* gene was significantly upregulated at baseline and treatment with rIFNα was followed by a significant downregulation of *PRDX2* in all MPNs.

In conclusion, our findings of a major impact of rIFNα upon several oxidative stress genes and antioxidative defense genes may imply an rIFNα mediated dampening of genotoxic damage to hematopoietic cells and stromal cells as well, thereby ultimately diminishing the risk of additional mutations that might drive the malignant clone towards myelofibrotic and leukemic transformation.

Having for the first time demonstrated rIFNα as a potent ROS modifying agent, our study has opened the avenue for further transcriptional studies to explore in depth the impact of stem-cell targeting therapy with rIFNα upon the epigenome, genome and proteome landscapes in MPNs. These studies should also include patients treated with hydroxyurea and combination therapies with anti-inflammatory agents, such as statins and JAK1-2 inhibitors. Such efforts may unravel novel important insights into the impact of such therapies on aberrant ROS-associated pathways to be targeted in the future [89, 90] and accordingly novel insights into the synergistic crosstalk between inflammation, oxidative stress, and genomic alterations [23].

## Supporting information

S1 Table The significantly most up- or downregulated genes in patients with ET, PV or PMF during treatment with rIFNα (data set 2). In addition, data from the same patients compared to controls (data set 2) and another cohort (data set 1) are presented. Ctrl: control; rIFNα: interferon-alpha2; ET: essential thrombocythemia; PV: polycythemia vera; PMF: primary myelofibrosis; FC: fold change; FDR: false discovery rate.
(XLSX)
